# Supplementary material for: A systematic review of causes of recent increases in ages of labor market exit in OECD countries
Source: PLoS One. 2020 Apr 29;15(4):e0231897. doi: 10.1371/journal.pone.0231897 (PMC7190130; doi:10.1371/journal.pone.0231897)
Supplement: S1 Table — (DOCX) [file pone.0231897.s001.docx]

**Table A1. Studies overview: data source and methods**

| **Author** | **Year** | **Journal** | **Data source** | **Year coverage** | **Model** | **Causality** | **Strategy for tracking causality** |
| --- | --- | --- | --- | --- | --- | --- | --- |
| Berkel & Börsch-Supan | 2004 | FinanzArchiv: Public Finance analysis | - German socio-economic panel | 1984-1997 | Probit | Yes | Option value  Social security wealth |
| Blau & Goodstein | 2010 | The Journal of Human Resources | - Current population survey - Survey of income and program participation - Social security administration | 1988-2005 | OLS | Yes | Calendar year fixed effects  Age fixed effects |
| Bönke et al. | 2018 | Labour Economics | - Insurance Account Sample | 1935-1945 | OLS | Yes | Inter-cohort variation in impact of reform  Structural dynamic retirement model |
| Buchholz et al. | 2013 | Comparative population studies | - German Socio-Economic Panel | 1934-1951 | Event-history models | Yes | Variation in introduction of new legislation across cohorts |
| Dejemeppe et al. | 2015 | IZA Journal of Labor Policy | - National social security office - National bank of Belgium | 2007-2008 | OLS | Yes | Macro-economic model  Forecast based on 1997-2007 period |
| Disney & Smith | 2002 | The Economic Journal | - British family expenditure survey | 1986-1994 | Probit-tobit | Yes | Differences-in-differences  Pre- and post-legislation effects  Younger and older age groups |
| Friedberg & Webb | 2005 | The Journal of Human Resources | - Health and Retirement Study | 1992-1998 | Probit | Yes | Peak value |
| Gustman & Steinmeier | 2009 | Research on ageing | - Current population survey - Health and Retirement Study | 1998-2004 | OLS | Yes | Structural econometric model  Inter-cohort variation in impact of reform |
| Hanel | 2010 | Labour Economics | - Insurance account sample | 1931-1942 | Proportional hazard | Yes | Age and cohort groups differently affected by reform |
| Hanel & Riphahn | 2012 | Labour Economics | - Swiss labor force survey | 2000-2005 | Logit | Yes | Differences-in-differences pre and post-legislation |
| Hurd & Rohwedder | 2011 | Journal of Population Ageing | - Current Population survey | 1992-2004 | Logit | Yes | Inter-cohrot change in proportion with DB/DC |
| Larsen & Pedersen | 2017 | Journal of Labour Market Research | - Eurostat - European Community health indicators - Survey of Health, Ageing and Retirement | 2004-2013 | Shift-share analysis | No | None |
| Mastrobuoni | 2009 | Journal of Public Economics | - Current population survey | 1928-1941 | OLS | Yes | Dynamic programming  Inter-cohort variation in phasing in reform |
| Puur et al. | 2015 | Post-Communist Economics | - Estonian national pension register | 2002-2011 | Averages | No | Difference before and after reform |
| Qi et al. | 2018 |  | - Swedish Interdisciplinary Panel | 1937-1944 | Dynamic programming | Yes | Inter-cohort variation in phasing in of reform |
| Pérez | 2020 | The Journal of the Economics of Ageing | - Spanish labor force survey | 1995-2016 | Probit | Yes | Simultaneous equations for effect of wives participation  Identification through inter-cohort increase in women's LFP |
| Schirle | 2008 | Journal of Labor Economics | - Current population survey - Canadian labor force survey - British quarterly labor force survey | 1994-2005 | Probit | Yes | Simultaneous equations for effects of wives’ participation  Identification through inter-cohort increase in women’s LFP |
| Staubli & Zweimüller | 2013 | Journal of Public Economics | - Austrian social security database | 2000-2010 | OLS | Yes | inter-cohort variation in phasing in reform |
| Staubli | 2011 | Journal of Public Economics | - Austrian social security database | 1991-2002 | Difference-in-difference | Yes | Differences-indifferences pre and post legislation |
