# Supplementary material for: A systematic review of causes of recent increases in ages of labor market exit in OECD countries
Source: PLoS One. 2020 Apr 29;15(4):e0231897. doi: 10.1371/journal.pone.0231897 (PMC7190130; doi:10.1371/journal.pone.0231897)
Supplement: S3 Table — (DOCX) [file pone.0231897.s003.docx]

**Table A3 Description of calculations, results reported in Figure 3**

| **Reference** | **Period** | **Observed values (in pp)** | **Observed change (in pp; yearly change* in parentheses)** | **Specification** | **Predicted values attributable to variable of interest (in pp; yearly change* in parentheses)** | **Variable of interest / area of change** |
| --- | --- | --- | --- | --- | --- | --- |
| Blau 2010 | 1998-1992 to 2001-2005** | 54.6 to 59.2 | 4.6 (**0.35**) | A  B  C  D  Average | 1.3  1.1  1.1  0.7  1.05 (**0.081**) | Delayed retirement credits |
|  |  |  |  | A  B  C  D  Average | 1.1  1.0  0.5  0.5  0.775 (**0.060**) | Normal retirement age |
|  |  |  |  | A  B  C  D  Average | 0.6  0.6  0.3  0.4  0.475 (**0.037**) | Average lifetime wealth |
|  |  |  |  | A  B  C  D  Average | 0.8  0.8  0.7  0.7  0.75 (**0.058**) | Spouse’s LFP |
|  |  |  |  | A  B  C  D  Average | 0.8  0.7  0.8  0.8  0.775 (**0.060**) | Educational attainment |
| Dejemeppe 2015 | 2007 to 2008.5 | 35.0 to 36.2 | 1.2 (**0.82**) | Men | 0.52 (**0.35**) | Extensive reform |
|  |  | 22.0 to 24.2 | 2.2 (**1.79)** | Women | 0.26 (0**.17**) |  |
| Larsen 2017 | 2004 to 2013 | 7.45 to 20.45 | 13.00 (**1.44**) | Men, Denmark | 1.17 (**0.13**) | Educational attainment |
|  |  | 5.04 to 5.48 | 0.44 (**0.05**) | Men, Germany | 0.10 (**0.01**) |  |
|  |  | 6.48 to 14.44 | 7.96 (**0.88**) | Men, Sweden | 1.43 (**0.16**) |  |
|  |  | 6.32 to 7.04 | 0.72 (**0.08**) | Women, Denmark | 0.45 (**0.05**) |  |
|  |  | 1.81 to 3.71 | 1.90 (**0.21**) | Women, Germany | 0.04 (**0.00**) |  |
|  |  | 3.43 to 7.29 | 3.86 (**0.43**) | Women, Sweden | 1.62 (**0.18**) |  |
| Pérez 2020 | 1995 to 2016 | 56.1 to 68.6 | 12.5 (**0.60**) | Men | 9.00 (**0.43**) | Partner’s LFP |
| Schirle 2008 | 1994 to 2005 | 67.0 to 73.4  66.8 to 73.2  66.9 to 73.3 | 6.4 (**0.58**) | 1 USA  2  Average | 2.4  2.3  2.35 (**0.21**) | Educational attainment |
|  | 1995 to 2005 | 60.9 to 68.1  61.0 to 68.3  61.0 to 68.2 | 7.3 (**0.72**) | 1 Canada  2  Average | 1.0  1.1  1.05 (**0.10**) |  |
|  | 1995 to 2005 | 64.8 to 71.4  65.6 to 71.9  65.2 to 71.7 | 6.5 (**0.65**) | 1 United Kingdom  2  Average | 0.9  1.0  0.95 (**0.10**) |  |
|  | 1994 to 2005 | 67.0 to 73.4  66.8 to 73.2  66.9 to 73.3 | 6.4 (**0.58**) | 1 USA  2  Average | 1.5  1.7  1.6 (**0.15**) | Spouse’s LFP |
|  | 1995 to 2005 | 60.9 to 68.1  61.0 to 68.3  61.0 to 68.2 | 7.3 (**0.72**) | 1 Canada  2  Average | 3.3  3.0  3.15 (**0.32**) |  |
|  | 1995 to 2005 | 64.8 to 71.4  65.6 to 71.9  65.2 to 71.7 | 6.5 (**0.65**) | 1 United Kingdom  2  Average | 2.3  1.7  2.0 (**0.20**) |  |

*Yearly change is calculated by dividing the value of interest by the length (in years) of the period of observation.

**Calculations refer to the middle point
