# Supplementary material for: A systematic review of causes of recent increases in ages of labor market exit in OECD countries
Source: PLoS One. 2020 Apr 29;15(4):e0231897. doi: 10.1371/journal.pone.0231897 (PMC7190130; doi:10.1371/journal.pone.0231897)
Supplement: S4 Table — Effects (i.e. regression coefficients) were extracted directly from articles where available and appear in column “Effect”. Estimates for separate groups appear in the columns “Value”; in such cases, it is the difference between groups that appears under “Effect”. (DOCX) [file pone.0231897.s004.docx]

**Table A4 Description of calculations, results reported in Figure 4.** Effects (i.e. regression coefficients) were extracted directly from articles where available and appear in column “Effect”. Estimates for separate groups appear in the columns “Value”; in such cases, it is the difference between groups that appears under “Effect”.

| **Reference** | **Reference group** | **Value** | **Treatment group** | **Value** | **Effect** | **Measurement** |
| --- | --- | --- | --- | --- | --- | --- |
| Gustman 2009 | Workers in year 1992 | “In main job”  Age 65: 15.9  Age 66: 13.0  Age 67: 10.8  “Full time work after retiring”  Age 65: 7.4  Age 66: 7.8  Age 67: 7.5  Sum (own calculations)  Age 65: 23.3  Age 66: 20.8  Age 67: 18.3 | Workers in year 2004 | “In main job”  Age 65: 17.3  Age 66: 14.0  Age 67: 11.6  “Full time work after retiring”  Age 65: 8.0  Age 66: 8.8  Age 67: 8.2  Sum (own calculations)  Age 65: 25.3  Age 66: 22.8  Age 67: 19.8 | Difference of sums (own calculations)  Age 65: 2.0  Age 66: 2.0  Age 67: 1.5 | Labor force participation (pp). |
| Hurd 2011 | Ages 51-54 in 1992 |  | Ages 51-54 in 2004 |  | Ages 57-60: 1.2  Ages 61-64: 2.1  Ages 65-68: 2.6 | Labor force participation (pp). |
| Staubli 2011 | Period 1994-1995 |  | Period 1998-1999 |  | Specification 1: 6.1  Specification 2: 7.5  Average (own calculation): 6.8 | Labor force participation (pp). |
| Staubli & Zweimüller 2013 | Men born before 1940 |  | Men born in 1940 or later |  | Specification 2  B. Employment: 9.8  C. Unemployment: 12.5  Sum (own calculation): 22.3 | Labor force participation (pp). |
|  | Women born before 1945 |  | Women born in 1945 or later |  | Specification 6  B. Employment: 11.0  C. Unemployment: 11.8  Sum (own calculation): 22.8 |  |
| Disney 2002 | Men ages 60-64 and 70-74 |  | Men ages 65-69 |  | 4.15 | Hours worked per week |
|  | Women ages 55-59 and 65-69 |  | Women ages 60-64 |  | 2.35 |  |
| Berkel 2004 | Men during the period 1984-1991 | Retirement age: 63.0 | Men during the period 1992-1997 | Retirement age: 61.2 | Difference in years (own calculation): 1.8 | Retirement age (in years) |
|  | Women during the period 1984-1991 | Retirement age: 24.4 | Women during the period 1992-1997 | Retirement age: 62.4 | Difference in years (own calculation): 0.7 |  |
| Hanel 2010 | Cohorts of men born in 1931-1936 | “Expected duration until employment exit” (in months from age 55):  57.7 | Cohorts of men born in 1937-1942 | “Expected duration until employment exit” (in months from age 55):  66.5 | Difference (in months): 8.81  Converted to years (own calculation): 0.73 | Retirement age (in years) |
|  | Cohorts of women born in 1931-1936 | “Expected duration until employment exit” (in months from age 55):  50.40 | Cohorts of women born in 1937-1942 | “Expected duration until employment exit” (in months from age 55):  49.85 | Difference (in months): -0.55  Converted to years (own calculation): -.05 |  |
| Bönke 2018 | Cohorts 1935-1936 |  | Cohorts 1939-1945 |  | Effect in months: 4.1  Converted to years (own calculation): 0.34 | Retirement age (in years) |
| Friedberg 2005 | Ages 53-57 in 1983 |  | Ages 53-57 in 2015 |  | Data source 1 (SCF): 9 months  Data source 2 (HRS): 13 months  Average (own calculation): 11 months  Converted to years (own calculation): 0.92 years | Retirement age (in years) |
| Qi 2018 | Men born in 1937 |  | Men born in 1944 |  | 0.15 years | Retirement age (in years) |
|  | Women born in 1937 |  | Women born in 1944 |  | 0.03 years |  |
| Mastrobuoni 2009 | Men born in 1928-1937 |  | Men born in 1938-1941 |  | 1.10 months  Converted to years (own calculation): 0.092 | Retirement age (in years) |
|  | Women born in 1928-1937 |  | Women born in 1938-1941 |  | 0.91 months  Converted to years (own calculation): 0.075 |  |
| Puur 2015 | Year 2002 | Mean retirement age: 57.6 | Year 2011 | Mean retirement age: 60.4 | Difference (own calculation): 2.8 years | Retirement age (in years) |
| Buchholz 2013 | Cohort 1934-1939 | Extracted from article:  Constant: -4.27  Ages 58-59: 0.65  Ages 60-61: 2.96  Ages 62-63: 2.61  Ages 64+: 3.64  Own calculations:  Constant + Age:  Ages 58-59: -3.62  Ages 60-61: -1.31  Ages 62-63: -1.66  Ages 64+: -0.63  exp(constant + age):  Ages 58-59: 0.027  Ages 60-61: 0.270  Ages 62-63: 0.190  Ages 64+: 0.534  exp(constant + age) / (1 + exp(constant + age)):  Ages 58-59: 0.026  Ages 60-61: 0.212  Ages 62-63: 0.160  Ages 64+: 0.348 | Cohort 1940-1945 | Extracted from article:  Effect birth cohort 1940-1945: -0.45  Own calculations:  Cohort 1940-45 + cohort 1934-39:  Ages 58-59: -4.07  Ages 60-61: -1.76  Ages 62-63: -2.1  Ages 64+: -1.08  exp(Cohort 1940-45 + cohort 1934-39):  Ages 58-59: 0.017  Ages 60-61: 0.172  Ages 62-63: 0.121  Ages 64+: 0.340  exp(Cohort 1940-45 + cohort 1934-39) / (1 + exp(Cohort 1940-45 + cohort 1934-39)):  Ages 58-59: 0.017  Ages 60-61: 0.147  Ages 62-63: 0.108  Ages 64+: 0.254 | Differences (own calculations):  Ages 58-59: 0.009  Ages 60-61: 0.066  Ages 62-63: 0.052  Ages 64+: 0.094 | Retirement probability (pp) |
|  |  |  | Cohort 1946-1951 | Extracted from article:  Effect birth cohort 1946-1951: -0.97  Own calculations:  Cohort 1940-45 + cohort 1934-39:  Ages 58-59: -4.59  Ages 60-61: -2.28  Ages 62-63: -2.63  Ages 64+: -1.60  exp(Cohort 1940-45 + cohort 1934-39):  Ages 58-59: 0.010  Ages 60-61: 0.102  Ages 62-63: 0.072  Ages 64+: 0.202  exp(Cohort 1940-45 + cohort 1934-39) / (1 + exp(Cohort 1940-45 + cohort 1934-39)):  Ages 58-59: 0.010  Ages 60-61: 0.093  Ages 62-63: 0.067  Ages 64+: 0.168 | Differences (own calculations):  Ages 58-59: 0.016  Ages 60-61: 0.120  Ages 62-63: 0.093  Ages 64+: .180 |  |
| Hanel & Riphahn 2012 | Period before 2001 |  | Period between 2001 and 2004 |  | Age 62: -0.239 | Retirement probability (pp) |
|  | Period between 2001 and 2004 |  | Period after 2004 |  | Age 62: -0.250 |  |
|  | Period between 2001 and 2004 |  | Period after 2004 |  | Age 63: -0.105 |  |
