# Supplementary material for: A systematic review of causes of recent increases in ages of labor market exit in OECD countries
Source: PLoS One. 2020 Apr 29;15(4):e0231897. doi: 10.1371/journal.pone.0231897 (PMC7190130; doi:10.1371/journal.pone.0231897)
Supplement: S1 Data — (DOCX) [file pone.0231897.s005.docx]

**Full strings used in searches**

**EconLit**

Search 1

TI (raise OR raising OR rise OR rising OR chang* OR increas* OR decreas* OR trend* OR grow* OR augment*) AND TI (labor force participation OR employment OR work OR labor force activity) AND TI (old OR older OR old age OR pension age OR older age OR retirement age OR elderly) AND LA English

Limiters: Published Date: 20000101-20191231; Publication Type: Journal Article.

Search 2

TI (raise OR raising OR rise OR rising OR chang* OR increas* OR decreas* OR trend* OR grow* OR augment*) AND TI (retirement age OR retirement timing OR retirement behavio* OR retirement decision) AND LA English

Limiters: Published Date: 20000101-20191231; Publication Type: Journal Article.

Search 3

TI (exten* OR prolong*) AND TI (working life) AND LA English

Limiters: Published Date: 20000101-20191231; Publication Type: Journal Article.

Search 4

TI (delay* OR postpon* OR late*) AND TI (retirement OR pension) AND LA English

Limiters: Published Date: 20000101-20191231; Publication Type: Journal Article.

**PubMed**
Search 1
 ((raise(Title) OR raising(Title) OR rise(Title) OR rising(Title) OR chang*(Title) OR increas*(Title) OR decreas*(Title) OR trend*(Title) OR grow*(Title) OR augment*(Title)) AND (labor force participation(Title) OR employment(Title) OR work(Title) OR labor force activity(Title)) AND (old(Title) OR older(Title) OR old age(Title) OR pension age(Title) OR older age(Title) OR retirement age(Title) OR elderly(Title))) AND (("2000"(Date - Publication) : "3000"(Date - Publication)) AND English(Language))

Search 2
((raise(Title) OR raising(Title) OR rise(Title) OR rising(Title) OR chang*(Title) OR increas*(Title) OR decreas*(Title) OR trend*(Title) OR grow*(Title) OR augment*(Title))) AND (retirement age(Title) OR retirement timing(Title) OR retirement behavio*(Title) OR retirement decision(Title)) AND (("2000"(Date - Publication) : "3000"(Date - Publication)) AND English(Language))

Search 3
((exten*(Title) OR prolong*(Title))) AND working life(Title) AND (("2000"(Date - Publication) : "3000"(Date - Publication)) AND English(Language))

Search 4
((delay*(Title) OR postpon*(Title) OR late*(Title))) AND (retirement(Title) OR pension(Title)) AND (("2000"(Date - Publication) : "3000"(Date - Publication)) AND English(Language))

**Web of science**

Search 1

(TI=((raise OR raising OR rise OR rising OR chang* OR increas* OR decreas* OR trend* OR grow* OR augment*) AND (labor force participation OR employment OR work OR labor force activity) AND (old OR older OR old age OR pension age OR older age OR retirement age OR elderly))) AND LANGUAGE: (English)

Indexes=SCI-EXPANDED, SSCI, A&HCI, ESCI Timespan=2000-2019

Search 2

(TI=((raise OR raising OR rise OR rising OR chang* OR increas* OR decreas* OR trend* OR grow* OR augment*) AND (retirement age OR retirement timing OR retirement behavio* OR retirement decision))) AND LANGUAGE: (English)

Indexes=SCI-EXPANDED, SSCI, A&HCI, ESCI Timespan=2000-2019

Search 3

(TI=((exten* OR prolong* ) AND (working life))) AND LANGUAGE: (English)

Indexes=SCI-EXPANDED, SSCI, A&HCI, ESCI Timespan=2000-2019

Search 4

(TI=((delay* OR postpon* OR late*) AND (retirement OR pension))) AND LANGUAGE: (English)

Indexes=SCI-EXPANDED, SSCI, A&HCI, ESCI Timespan=2000-2019
