# Supplementary material for: A systematic review of causes of recent increases in ages of labor market exit in OECD countries
Source: PLoS One. 2020 Apr 29;15(4):e0231897. doi: 10.1371/journal.pone.0231897 (PMC7190130; doi:10.1371/journal.pone.0231897)
Supplement: Attachment — Submitted filename: letter to authors.pdf [file pone.0231897.s006.pdf]

Report on the paper: "A systematic review of causes of recent increases in ages of labor market exit in OECD countries"

Summary and overall appraisal

According to the authors, the aim of the paper is, on the one hand, to provide an overview of the retirement domains into which current evidence falls, thus identifying research gaps; and, on the other hand, to assess the proportion of change in ages of labor market exit attributable to each investigated cause.

The paper does not have a high technical complexity from a statistical point of view. In fact they do not carry out any statistical analysis by themselves and their conclusions rely on the statistical analysis (mainly econometric analysis) from other research works.

Although the topic studied is relevant and hence worthy of investigation, currently the paper lacks some important issues that should be addressed in a future version of it, in order to reach a publishable standard, from my point of view. Thus, I encourage the authors to take into account seriously the comments detailed below.

Comments

1. As the own authors point out, one of their basic goals is to perform a "systematic review". In this vein, I miss some relevant references on this literature, for example:

> Pérez, C., Martín-Román, Á., & Moral, A. (2015). The impact of leisure complementarity on the labour force participation of older males in Spain. *Applied Economics Letters*, *22*(3), 214-217.

The authors should also check some of the references contained in it. Another interesting paper for a country not belonging to the OECD, but containing literature on this topic is:

> Queiroz, B. L., & Souza, L. R. (2017). Retirement incentives and couple's retirement decisions in Brazil. *The Journal of the Economics of Ageing*, *9*, 1-13.

2. In addition, there is a fresh paper investigating thoroughly one of the domains that the authors identify. Despite being published after the bibliographical database was collected (February 2019), I think it could be also included in a revised version of the paper:

> Pérez, C., Martín-Román, Á., & Moral, A. (2019). Two decades of the complementary leisure effect in Spain. *The Journal of the Economics of Ageing*, forthcoming.

3. Tables 4 and 5 constitute the core of the paper. The authors carry out a descriptive study by reviewing some of the literature on the topic. However, I am afraid that they are comparing a set of very heterogeneous results. I think no clear conclusion can be obtained by just looking at these numbers without a quantitative treatment of them. Particularly, I deem that with such analysis they cannot "*assess the proportion of change in ages of labor market exit attributable to each investigated cause*" as they state when defining their goals.

4. Following the argument elaborated in my previous comment, I would suggest the authors to perform a meta-analysis. The information contained in tables 4 and 5 might be a promising starting point to do that.

5. I would also suggest the authors to review the writing and to rewrite some parts of paper in a more structured manner.
