# Supplementary material for: A systematic review of causes of recent increases in ages of labor market exit in OECD countries
Source: PLoS One. 2020 Apr 29;15(4):e0231897. doi: 10.1371/journal.pone.0231897 (PMC7190130; doi:10.1371/journal.pone.0231897)
Supplement: Attachment — Submitted filename: letter to editor.pdf [file pone.0231897.s007.pdf]

Dear editor,

My recommendation regarding the paper entitled: "***A systematic review of causes of recent increases in ages of labor market exit in OECD countries***" is rejection. The reason for that is that, in my view, the paper does not fulfill any of the two claimed main goals.

First, I think that the review is not so "systematic". Eighteen papers about this topic is not a very impressive number. Moreover, I miss some relevant references on this literature. Second, I do not believe that their descriptive analysis let them conclude that they are assessing "the proportion of change in ages of labor market exit attributable to each investigated cause", as they state. In my opinion, the should carry out some type of quantitative analysis to control for some differences in the examined studies and, in this way, to avoid biased comparisons among highly heterogeneous results found in the reviewed papers. Ideally, I suggest them to perform a meta-analysis.

Best regards,

Ángel Martín-Román
