# Supplementary material for: A systematic review of causes of recent increases in ages of labor market exit in OECD countries
Source: PLoS One. 2020 Apr 29;15(4):e0231897. doi: 10.1371/journal.pone.0231897 (PMC7190130; doi:10.1371/journal.pone.0231897)
Supplement: Attachment — Submitted filename: review_comments.pdf [file pone.0231897.s009.pdf]

Reviewer #2 – Comments

Summary and overall appraisal
According to the authors, the aim of the paper is, on the one hand, to provide an overview of the retirement domains into which current evidence falls, thus identifying research gaps; and, on the other hand, to assess the proportion of change in ages of labor market exit attributable to each investigated cause.

The paper does not have a high technical complexity from a statistical point of view. In fact they do not carry out any statistical analysis by themselves and their conclusions rely on the statistical analysis (mainly econometric analysis) from other research works.

Although the topic studied is relevant and hence worthy of investigation, currently the paper lacks some important issues that should be addressed in a future version of it, in order to reach a publishable standard, from my point of view. Thus, I encourage the authors to take into account seriously the comments detailed below.

As the own authors point out, one of their basic goals is to perform a "systematic review". In this vein, I miss some relevant references on this literature, for example:

Pérez, C., Martín-Román, Á., & Moral, A. (2015). The impact of leisure complementarity on the labour force participation of older males in Spain. *Applied Economics Letters*, *22*(3), 214-217.
The authors should also check some of the references contained in it. Another interesting paper for a country not belonging to the OECD, but containing literature on this topic is:
Queiroz, B. L., & Souza, L. R. (2017). Retirement incentives and couple's retirement decisions in Brazil. *The Journal of the Economics of Ageing*, *9*, 1-13.

2. In addition, there is a fresh paper investigating thoroughly one of the domains that the authors identify. Despite being published after the bibliographical database was collected (February 2019), I think it could be also included in a revised version of the paper:
Pérez, C., Martín-Román, Á., & Moral, A. (2019). Two decades of the complementary leisure effect in Spain. *The Journal of the Economics of Ageing*, forthcoming.

3. Tables 4 and 5 constitute the core of the paper. The authors carry out a descriptive study by reviewing some of the literature on the topic. However, I am afraid that they are comparing a set of very heterogeneous results. I think no clear conclusion can be obtained by just looking at these numbers without a quantitative treatment of them. Particularly, I deem that with such analysis they cannot "*assess the proportion of change in ages of labor market exit attributable to each investigated cause*" as they state when defining their goals.
4. Following the argument elaborated in my previous comment, I would suggest the authors to perform a meta-analysis. The information contained in tables 4 and 5 might be a promising starting point to do that.
5. I would also suggest the authors to review the writing and to rewrite some parts of paper in a more structured manner

Reviewer # 3 - Comments:

I appreciate the authors' receptiveness to earlier critiques and their inclusion of changes. However, I still have a concern about the results in Figure 4, and what these estimates are – I'm afraid they still may be confusing coefficient estimates with explanations of historical variation.

For instance, their footnote mentions "or example, in a study by Berkel among men in Germany, the effect of treatment (pension reform) measured as a difference in retirement age between treatment and control group was 1.8 year." This implies that the coefficient estimate from the study was 1.8 years. This does *not* mean that the study concluded that 1.8 percentage points of change in LFP was attributable to the treatment.

Additionally, in referring to the Staubli and Zweimuller paper, the authors seem to imply that 22.8 percentage points and 22.3 percentage points of the total change in LFP is attributable to early retirement changes. However, after looking at the PMC-available version of this paper (available here: https://www.ncbi.nlm.nih.gov/pmc/articles/PMC3851570/pdf/nihms-526007.pdf), I'm unable to figure out how the authors obtained this 22.8 and 22.3, especially since according to Table 2 of the Staubli and Zweimuller paper, the employment rate for the sample seemed to only change by 19 percentage points for men and 25.4 percentage points for women. The Staubli and Zweimuller paper doesn't seem to have any sort of breakdown of how much of the historical change was explained by their results, which is what the authors seem to imply that the 22.8 and 22.3 represent – the paper seems concerned with the ceteris paribus effects of a policy change rather than an explanation of historical variation. I've only probed into this one paper, but I'm concerned that this would apply to the other papers that the authors reference, especially in light of the footnote describing the Berkel paper.
