# Supplementary material for: A systematic review of causes of recent increases in ages of labor market exit in OECD countries
Source: PLoS One. 2020 Apr 29;15(4):e0231897. doi: 10.1371/journal.pone.0231897 (PMC7190130; doi:10.1371/journal.pone.0231897)
Supplement: Attachment — Submitted filename: round2_reply_reviewers_final.docx [file pone.0231897.s010.docx]

We would like to thank the editor and three anonymous reviewers for their attention to the changes that we brought to the last version of the manuscript and for the new comments that they provided. Below we answer each comment one-by-one and indicate whether each of them led to changes in the manuscript.

**Editor**

1. **In general, there is an disagrement by how  systematic is the review. There are some  miussing relevant references on this literature.**

Unfortunately, we cannot increase the number of articles reviewed, because this number depends on the review protocol that we had previously established, which stated among others the search strategy and inclusion criteria. We thank the reviewers for suggesting articles for inclusion. The inclusion of these articles was assessed based on the same criteria as all other articles included in the review. One article suggested in the previous round satisfied our criteria, and was subsequently included in the review, but the other ones did not satisfy our criteria, and were therefore not included. We discuss this point in more detail under the second reviewer’s comments #1 and #4, including the reasons why the recommended articles were not included.

2. **the descriptive analysis do not lead to the conclude that the paper  assess “the proportion of change in ages of labor market exit attributable to each investigated cause”,**

We have revised the introduction section and rephrased the objective of our study. The original objective stated that we assessed “the proportion of change in ages of labor market exit attributable to each investigated cause”. The new objective states that we “compare studies with respect to the magnitude of change in retirement behavior that they attributed to different causes, in different contexts.”

3. **one suggestions is to incorporate a broader literature and perform a meta-analysis**

As explained above, based on our pre-established criteria, there are no more articles to be included in our review.

Concerning the meta-analysis, we believe that the current analysis of the results is more optimal than a meta-analysis. In short, there are many different classes of predictors and outcomes in the papers reviewed. Because of this, we would need to perform meta-analyses on very small clusters of articles (most often 2 or 3). Furthermore, even when predictors and outcomes are comparable, the context in which their association was estimated usually differs considerably. Also, an important finding is that there is considerable heterogeneity in the results arrived at in the articles reviewed. Therefore, we do not find it advisable to include outcomes in a single regression model, which would mask this heterogeneity. We elaborate more on this point under Reviewer’s 2 comment #2.

4. **the paper does not carry out any statistical analysis but, the conclusions rely on the statistical analysis. So, we believe this is an important adjustment to the paper.**

Our paper indeed does not carry out any original statistical analysis. The analytical part is limited to re-calculation of the results from original studies to provide comparability between them. We revised our manuscript to avoid suggesting that our conclusions are based on statistical analyses. We elaborate on this under the Second reviewer’s comment #3.

5. **The detailed comments by Reviewer #2 and # 3 are organized in the attached document.**

We address each comment below.

**Reviewer #1**

**I am happy with the revised version of the paper. I like the research question, the structure of the paper, the quality of writing, and the way the authors describe their empirical proceeding and results. Most importantly, the authors have addressed all the issues stated in my referee report for the first version appropriately.**

We thank reviewer 1 for their appreciation of the changes we brought to the manuscript.

**Reviewer #2**

**First of all, I would like to acknowledge that the authors have carried out an important review work. Thus, I would like to congratulate them for that. I strongly believe that the paper has improved significantly in this second round. However, I still have some important remarks to make.**

**1. Now I understand the label “systematic review”. The authors explain that it is the name of a research technique. In any case, the number of articles used is still very small to draw any sensible “general” conclusion. Despite the fact that they make use of arguments from authority to justify this sample size, I cannot see how they can make any inference for the OECD countries. Particularly with such an unbalanced sample of countries (e.g. six studies for Germany and so on). I align myself here with reviewer 1.**

We agree with Reviewer 2 that the studies reviewed in our article cover a relatively limited number of countries. Eleven of 36 OECD countries are covered, but many of the findings come from studies analyzing US or German data. We modified the manuscript to clarify that we limit our review to OECD countries, since they are in comparable phases of population aging and trends in ages of labor market exit, but that our conclusions only apply to those countries for which evidence was available, and cannot be generalized for all OECD countries. We also mention the fact that Germany and the United States are overrepresented as a limitation. We also stress that future research should concentrate on more countries.

**2. As for the quantitative analysis, the authors claim that “a meta-analysis was not feasible for this article”. I do not agree with that. It is true, however, that they should define an appropriate dependent variable (this the first and key decision to be made) and a set of independent variables, many of them defined as dummy variables. In order to discard the meta-analysis, they state that “their independent variables denoted varying realities”, “studies included had various designs” and “methodologies varied considerably too”. I wonder if such “flaws” do not impede to perform a wise qualitative analysis. My point of view is different: a good meta-analysis could overcome that issues.**

A meta-analysis combines results from different studies to provide more precise estimates of an association between two variables. A regression model is estimated based on estimates of the outcome of interest and the associated standard errors retrieved from each reviewed article. Hence, to perform a meta-analysis, we would have needed to define an association for which we wish to have more precise estimates. As shown in Table 3, studies included in our review measured change in retirement behavior based on four types of outcomes, and change in different retirement domains were based on ten different types of predictors. In total, 19 combinations of outcomes and predictors were found. Eleven of these combinations were covered by only one study, meaning these combinations could not, by definition, be included in a meta-analysis. The other eight combinations were covered by two to four studies. As a result, we could have at best presented results from eleven studies taken separately, together with results of meta-analyses performed on eight groups of two to four studies. We believe such an approach would lead to highly scattered results, and add very limited added value to the systematic review.

Another problem limiting the prospect of a meta-analysis was that we would have needed to estimate models based on estimates obtained in largely different contexts. For example, effects of changes in early retirement schemes found in Belgium in 2004-2013 (Dejemeppe et al. 2015) would have been estimated alongside effects found in Austria in 1994-1999 (Staubli 2011). Since context matters considerably when comparing the effects of policy reforms, we found it more advisable not to combine such results in a regression model and focus on a substantial interpretation.

In sum, because of the large number of combinations on which to estimate models, and because estimates were obtained in varying contexts, we choose not to perform a meta-analysis on the results found in the studies that we reviewed. We think our current approach, offering a more qualitative analysis based on the different combinations of predictors and (different types of) outcome variables, while considering the specific context of the studies, offers a better basis for conclusions.

**3. As regards the quantitative analysis again, the authors allege to have performed some quantitative analysis. I do not think so. For instance, they state: “quantitative results were originally presented in tables”. In my view, what they call quantitative results are only some figures extracted from other research works. I do not want to appear as a maximalist quantitative researcher, but the truth is that PLOS ONE is a journal that specifically encourages the use of a sound statistical analysis. In fact, one of the questions I have to answer as a reviewer is: “Has the statistical analysis been performed appropriately and rigorously?” To be honest there is a lack of statistical analysis. There is only a use of statistics from a descriptive perspective.**

We agree with the reviewer that we did not perform any original statistical analysis in our article. However, according to the PLOS ONE author guidelines, this is not necessary when performing a systematic review: “A systematic review paper, as defined by The Cochrane Collaboration, is a review of a clearly formulated question that uses explicit, systematic methods to identify, select, and critically appraise relevant research, and to collect and analyze data from the studies that are included in the review. These reviews differ substantially from narrative-based reviews or synthesis articles. Statistical methods (meta-analysis) may or may not be used to analyze and summarize the results of the included studies” (Plos One Submission Guidelines, accessed online on the 19^th^ of March 2020 under <https://journals.plos.org/plosone/s/submission-guidelines>).

We have explained above why we chose not to apply our own statistical analysis (meta-analysis). Instead, we extracted figures from the reviewed articles and re-calculated them to compare the results across different causes of increases in retirement ages and different contexts. To avoid confusion, we reformulated the goal of our study in the introduction section, which now reads “To compare studies with respect to the magnitude of change in retirement behavior that they attributed to different causes, in different contexts”.

**4. After reading the authors’ response to my first comment in the previous round, I am seriously questioning the inclusion criteria followed. If Perez et al. (2015) does not qualify for inclusion, that criteria should be revised. I affirm this because this article practically mimics the work by Schirle (2008), which is included.**

We considered the article from Perez et al. (2015) thoroughly, but could not include it because it did not adhere to the inclusion criteria. More specifically, as specified in the manuscript, selected studies should explain *changes over time* in labor force participation / ages of labor market exit. The study of Perez et al. (2015) assesses the determinants of labor force activity among men ages 55-64, focusing on the propensity of wives to participate in the labor market. The study finds that older men are more likely to participate if their wives also do so. However, the study does not measure how much of the increase over time in men’s labor force participation is due to increases over time in women’s participation, in contrast to Schirle (2008).

**Reviewer #3**

**I would supplement the data included in the paper with exactly what point estimates and what results were used from each paper.**

We agree with this point from reviewer 3 and added the point estimates and results used from each paper to the appendix.

**I appreciate the authors’ receptiveness to earlier critiques and their inclusion of changes. However, I still have a concern about the results in Figure 4, and what these estimates are – I’m afraid they still may be confusing coefficient estimates with explanations of historical variation.**

**For instance, their footnote mentions “or example, in a study by Berkel among men in Germany, the effect of treatment (pension reform) measured as a difference in retirement age between treatment and control group was 1.8 year.” This implies that the coefficient estimate from the study was 1.8 years. This does not mean that the study concluded that 1.8 percentage points of change in LFP was attributable to the treatment.**

We fully agree with reviewer 3 that the study by Berkel identifies a coefficient estimate of 1.8 years and that the study did not conclude that 1.8 percentage points of change in LFP was attributable to the treatment. However, this is also what we attempted to summarize in the article.

As for the possible confusion between explanations of historical variation and coefficient estimates, Figure 3 presents results from articles that provided explanations of historical variation, while Figure 4 provides coefficients estimates (between groups). We went through the manuscript again to make sure that we did not mix up the two types of results. We adjusted the wording in the method and results sections, including the description of the figures, to avoid confusion as much as possible.

**Additionally, in referring to the Staubli and Zweimuller paper, the authors seem to imply that 22.8 percentage points and 22.3 percentage points of the total change in LFP is attributable to early retirement changes. However, after looking at the PMC-available version of this paper (available here: https://www.ncbi.nlm.nih.gov/pmc/articles/PMC3851570/pdf/nihms-526007.pdf), I’m unable to figure out how the authors obtained this 22.8 and 22.3, especially since according to Table 2 of the Staubli and Zweimuller paper, the employment rate for the sample seemed to only change by 19 percentage points for men and 25.4 percentage points for women. The Staubli and Zweimuller paper doesn’t seem to have any sort of breakdown of how much of the historical change was explained by their results, which is what the authors seem to imply that the 22.8 and 22.3 represent – the paper seems concerned with the ceteris paribus effects of a policy change rather than an explanation of historical variation. I’ve only probed into this one paper, but I’m concerned that this would apply to the other papers that the authors reference, especially in light of the footnote describing the Berkel paper.**

We are comparing articles according to labor force participation. The values of 22.8 and 22.3 percentage points were obtained by adding the effects of covariates on employment (11.00 pp; 9.75 pp) and unemployment (11.77 pp; 12.51 pp), this is 11.00 + 11.77 = 22.8 and 9.75 + 12.51 = 22.3. The original figures can be found in columns 2 and 6 of Table 3, page 25 of Staubli and Zweimuller (2013). We provide more detailed information in the new appendix that shows how each of the value in the Figures was obtained. Once again, we reviewed the manuscript to make sure that results presented in Figure 3 refer to explanations of historical trends, and that results presented in Figure 4 refer to coefficient estimates, and not vice-versa.
